# Arthrogryposis Multiplex Congenita: Multiple Congenital Joint Contractures

**DOI:** 10.1155/2015/379730

**Published:** 2015-10-28

**Authors:** Hamza Sucuoglu, Nurettin Irem Ornek, Cagkan Caglar

**Affiliations:** ^1^Private Bagcilar Medicine Center, Department of Physical Medicine and Rehabilitation, 34204 Istanbul, Turkey; ^2^Private Safir Medicine Center, Department of Physical Medicine and Rehabilitation, 34265 Istanbul, Turkey

## Abstract

Arthrogryposis multiplex congenita (AMC) is a syndrome characterized by nonprogressive multiple congenital joint contractures. The etiology of disease is multifactorial; it is most commonly suspected from absent fetal movements and genetic defects. AMC affects mainly limbs; also it might present with other organs involvement. It is crucial that the diagnosis of AMC should be kept in mind by musculoskeletal physicians in newborns with multiple joint contractures and patients must begin rehabilitation in early stage after accurate diagnosis in terms of functional independence. We present the diagnosis, types, clinical features, and treatment approaches of this disease in our case with literature reviews.

## 1. Introduction

Arthrogryposis multiplex congenita (AMC) is a syndrome characterized by nonprogressive multiple congenital joint contractures. AMC is actually a common clinical name caused by many different syndromes rather than a single syndrome [1]. The etiology of the disease is unclear. Although several factors are accused, most frequently the absence of fetal movement is shown as the cause of the disease [2–5]. AMC affects mainly the extremities and it might also be involved in the other organs [2, 4]. There are two main types of the disease. Amyoplasia is the most common (40%) (classical) type. The second type is distal arthrogryposis. Types, clinical features, locations of genetic defect, and numbers of phenotypes of distal arthrogryposis syndrome have been particularly described in Online Mendelian Inheritance in Man (OMIM) (Table 1) [6]. The main involvement is contractures of joints in both of these two types [1, 6, 7]. The correct diagnosis and the initiation of appropriate physical medicine and rehabilitation (PMR) program in a multidisciplinary approach, in terms of the patient's functional independence and ambulation in AMC, are crucial. In light of our AMC case, we will discuss the diagnosis, types, treatment, and rehabilitation approaches of this disease with literature reviews.

## 2. Case

**Figure 1:**
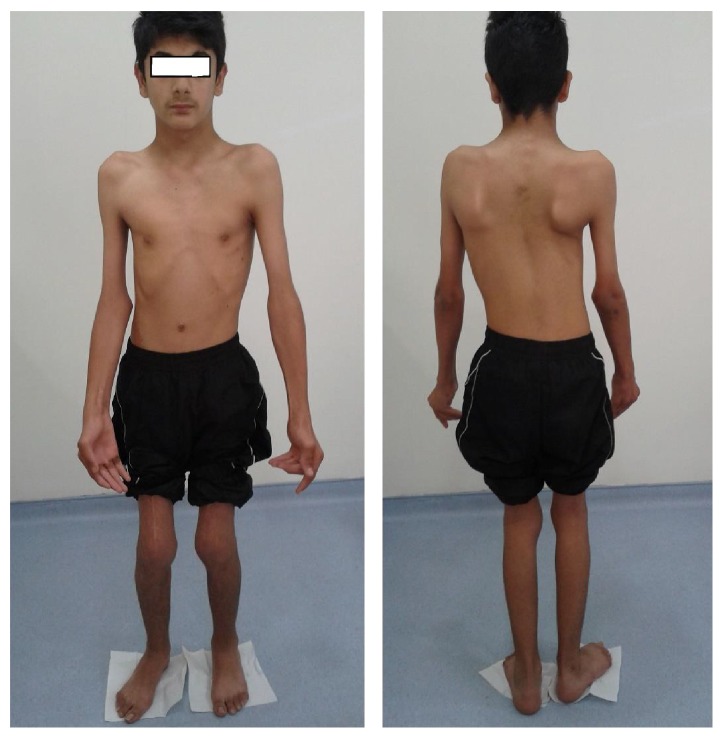
There were ptosis of the left eyelid, generalized muscle atrophy, weak body parts, webs/membranes (pterygium) of the neck, elbows, wrists, and fingers, semiflexed position in the elbows and knees, pes equinovalgus deformity of the right foot, flattening of the lumbar lordosis, C-type scoliosis with the thoracic curve to the right, and winged scapula.

**Figure 2:**
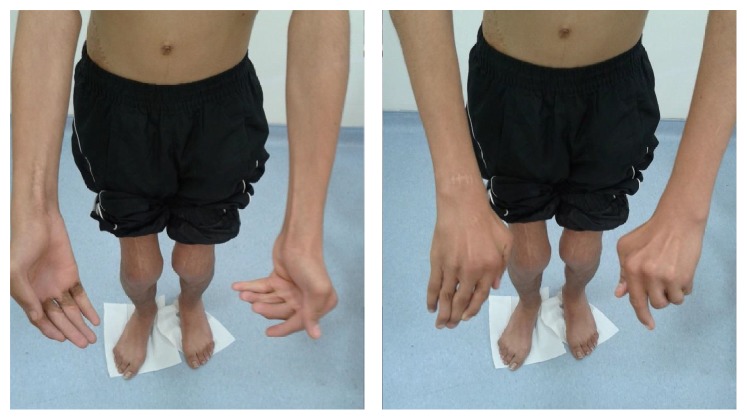
There were ulnar deviations in the fingers and flexion contractures in the proximal and distal interphalangeal joints of both hands. Fingers were thinner and longer than normal.

A thirteen-year-old male patient was admitted to the outpatient clinic with complaints of common deformity in joints, movement restrictions, and difficulty walking. Patient had complaints of joint pain since he was born and he was the second son of his family. The first child who was the brother of the patient was healthy. He was diagnosed as having AMC after his birth because of the characteristic appearance and multiple contractures of the fingers. PMR programs were applied in several centers previously. He had undergone a soft-tissue release operation of both knees, lengthening operation of the quadriceps tendon, and extending operation of the right wrist and finger flexor muscle group when he was 3 years old. On examination there were ptosis of the left eyelid, poorly defined conchae in ears, generalized muscle atrophy, weak body parts, webs (pterygium) of the neck, elbows, wrists, and fingers, semiflexed position in the elbows and knees, flattening of the lumbar lordosis, C-type scoliosis, and winged scapula (Figure 1). The knees were slightly flexed and he was able to ambulate independently. Findings of range of motion were noted as 160 degrees for flexion and abduction, 80 degrees for internal and external rotator at bilateral shoulders, 20 degrees for extension and 70 degrees for flexion at bilateral wrists, 20 degrees for flexion at right knee, 30 degrees for flexion at left knee, and 10 degrees for extension at bilateral knees. There were flexion contractures in the proximal and distal interphalangeal joints of both hands (Figure 2). Neurological exam was normal. Laboratory tests were normal. Patient was included in PMR program. Stretching and passive ROM exercises for contractures, scoliosis and posture exercises, and active resistive extremity-strengthening, balance-proprioceptive, and walking exercises were performed. Thoracolumbar orthosis for scoliosis was given. Hot pack and therapeutic ultrasound were given to the contracted muscles before stretching exercises and electrostimulation applications were performed to increase the strength of related muscles. PMR program lasted for 30 sessions. Improvements of 10–15 degrees in passive ROM measurements of both shoulders and knees and improvements in gait and balance functions have been obtained. There was no significant scoliosis curve improvement. We proposed close follow-up and orthopedic outpatient clinic control. Additionally, during and after the treatment, a home-based exercise program was taught to family caregivers.

## 3. Discussion

Arthrogryposis multiplex congenita (AMC) is defined as a common clinical name caused by many different syndromes which can be accompanied by the findings from other systems and characterized by congenital soft-tissue contractures in multiple joints [1].

Although the etiology of disease is not clear, many genetic defects are shown in the pathogenesis of AMC. These may be due to single-gene mutations, chromosomal abnormalities, and mitochondrial defects [2, 4]. It commonly occurs in one of every 3000 live births [8]. AMC is diagnosed prenatally by ultrasound or during birth [4]. Our case was sporadic and history of family was normal. The diagnosis had been made just after the birth due to characteristic multiple joint contractures. Although a large number of AMC forms have been defined, it is essentially classified as limb involvement or limb involvement with the other organ involvement [6, 9].

Amyoplasia is a sporadic condition and the intelligence is normal and there is no major malformation in the central nervous system, heart, gastrointestinal tract, and genitourinary system in patient with amyoplasia [2, 10]. In these cases, all four limbs are involved in a symmetrical pattern. Extension contracture of the elbow and deformities of flexion and ulnar deviation at wrist are common. There is hooklike appearance of the wrist and fingers due to metacarpophalangeal and interphalangeal joint flexion contractures [11]. In our case, there were contractures of shoulders, wrists, hand fingers, and knees. There were ulnar deviation of the fingers and the hook appearance of the hand. There was no pathology in the other visceral system. Although the main problem is multiple joint contractures in AMC, it may be accompanied by the other symptoms. These can include thin skin, muscle atrophies, limb anomalies (such as shortness, webs, radial head dislocations, and patellar aplasia), abnormalities of face and jaw (asymmetry, depressed nasal bridge, micrognathia, trismus, and hemangioma), scoliosis, and different anomalies of respiratory, urinary, and nervous systems. In this case, patient's symptoms were not only multiple contractures of joints, but also scoliosis, ptosis, pterygiums (on neck, elbows, wrists, and fingers), and muscular hypoplasia. Treatment must be planned according to the patient; long-term rehabilitation programs at home should be explained to the patients and caregivers [5]. Multidisciplinary approach is essential for the treatment; splints or serial casting, passive stretching, and range of motion exercises are particularly effective in the treatment of contractures [2, 12]. Although patients of the classical arthrogryposis gain back their independence in their activities of daily living some patients may need help from other people even in adulthood [3, 5]. Also early surgical release of contracted tissue of joints which constitutes the basis of the problem is crucial for the prevention of deformity [13]. He had continued the rehabilitation program in several rehabilitation units since being diagnosed in early childhood and also he had undergone release operations for the contractures when he was 3 years old. Stretching and passive range of motion exercises for contractures and scoliosis were performed and also balance and walking exercises were performed to improve the scoliosis, balance, and gait problems. The functional capacity of the independently ambulatory patients was further improved with this treatment program. Progressive scoliosis which is one of the other significant problems occurs in the great majority of patients with AMC disease [1, 2, 14]. Radiological follow-up of the scoliosis should be performed regularly and surgery is currently recommended when curve magnitude exceeds 40 degrees [15]. We treated our patient with corset treatment and regular exercise. However there were no signs of improvement so we recommended continuing scoliosis home exercise program, close follow-up, and orthopedic consultation.

## 4. Conclusion

In patients with AMC, physical therapy program should be started in the early stage after being diagnosed. The goals of AMC treatment include being able to walk and maintaining the activities of daily living independently. In this respect, passive stretching exercises, splints, serial casting, and release surgery are of great importance in the treatment of AMC.

## Figures and Tables

**Table 1:** Syndrome types, labels, and OMIM Numbers for the distal arthrogryposis^*∗*^.

Syndrome	New label	OMIM Number
Distal arthrogryposis type 1	DA1	108120
Distal arthrogryposis type 2A (Freeman-Sheldon syndrome)	DA2A	193700
Distal arthrogryposis type 2B (Sheldon-Hall syndrome)	DA2B	601680
Distal arthrogryposis type 3 (Gordon syndrome)	DA3	114300
Distal arthrogryposis type 4 (scoliosis)	DA4	609128
Distal arthrogryposis type 5 (ophthalmoplegia, ptosis)	DA5	108145
Distal arthrogryposis type 6 (sensorineural hearing loss)	DA6	108200
Distal arthrogryposis type 7 (trismus-pseudocamptodactyly)	DA7	158300
Distal arthrogryposis type 8 (autosomal dominant multiple pterygium syndrome)	DA8	178110
Distal arthrogryposis type 9 (congenital contractural arachnodactyly)	DA9	121050
Distal arthrogryposis type 10 (congenital plantar contractures)	DA10	187370

^*∗*^OMIM = Online Mendelian Inheritance in Man.

## References

[1] Kalenderer Ö., Önvural B. (2009). Artrogripozis multipleks konjenita. *TOTBİD Dergisi*.

[2] Baser O. C., Ay S., Dogan S. K., Evcik D. (2009). Multipl konjenital eklem kontraktürleri, artrogripozis multipleks konjenita: Bir olgu sunumu. *Turkish Journal of Rheumatology*.

[3] Cinar C., Sezgin M., Aydog E., Cakci A. (2004). Multipl konjenital kontraktürler (artrogripozis multipleks konjenita). *Turkish Journal of Rheumatology*.

[4] Hall J. G. (1997). Arthrogryposis multiplex congenita: etiology, genetics, classification, diagnostic approach, and general aspects. *Journal of Pediatric Orthopaedics—Part B*.

[5] O'Flaherty P. (2001). Arthrogryposis multiplex congenita. *Neonatal Network*.

[6] Bamshad M., Van Heest A. E., Pleasure D. (2009). Arthrogryposis: a review and update. *The Journal of Bone and Joint Surgery—American Volume*.

[7] Gökkaya N. K. O., Uçan H., Uçkun A. Ç., Alanay Y. (2011). Beals Hecht syndrome-arthrogryposis multiplex congenita-congenital arachnodactyly: case report. *Turkiye Fiziksel Tip ve Rehabilitasyon Dergisi*.

[8] Fahy M. J., Hall J. G. (1990). A retrospective study of pregnancy complications among 828 cases of arthrogryposis. *Genetic Counseling*.

[9] Hall J. G. (1981). An approach to congenital contractures (arthrogryposis). *Pediatric Annals*.

[10] Sarwark J. F., MacEwen G. D., Scott C. I. (1990). Amyoplasia (a common form of arthrogryposis). *The Journal of Bone & Joint Surgery—American Volume*.

[11] Ardic F., Yorgancioglu Z. R., Kahraman Y., Findikoglu G. (2003). Artrogripozisde değerlendirme ve rehabilitatif yaklaşım. *Turkish Journal of Rheumatology*.

[12] Herring J. A. (2002). *Tachdjian's Pediatric Orthopaedics*.

[13] Palmer P. M., MacEwen G. D., Bowen J. R., Mathew P. A. (1985). Passive motion therapy for infants with arthrogryposis. *Clinical Orthopaedics and Related Research*.

[14] Beals R. K., Hecht F. (1971). Congenital contractural arachnodactyly. A heritable disorder of connective tissue. *The Journal of Bone & Joint Surgery—American Volume*.

[15] Weinstein S. L., Weinstein S. L. (2001). Marfan syndrome. *The Paediatric Spine. Principles and Practice*.

